# Navigating heavy metal stress: emerging roles of TOR and SnRK signaling in plant tolerance

**DOI:** 10.3389/fpls.2026.1774622

**Published:** 2026-06-03

**Authors:** Sheeba Naaz, Ashverya Laxmi

**Affiliations:** National Institute of Plant Genome Research, New Delhi, India

**Keywords:** heavy metals, signaling, SnRK, stress tolerance, TOR, TOR–SnRK crosstalk

## Abstract

This review explores emerging insights about how plants regulate their responses to heavy metal stress through the coordinated actions of the Target of Rapamycin (TOR) and Sucrose Non-Fermenting-1-Related Kinase (SnRK) signaling pathways. Toxic heavy metals such as As, Pb, Cd and Hg cause severe metabolic and oxidative stress in plants, which reduces their growth and development and ultimately disrupts cellular homeostasis. In this review, we highlight the unique direction of research that focuses on TOR–SnRK interaction under heavy metal exposure, emphasizing their opposite yet interconnected roles in metabolic reprogramming, stress tolerance, and in growth regulation. Under heavy metal stress, SnRK kinases are activated, which triggers the expression of stress-responsive genes and activates autophagy, while downregulating TOR activity to conserve energy and divert resources toward defense, which maintains redox homeostasis, allows plants able to survive. TOR–SnRK pathways interacts with calcium, hormonal, and redox signaling networks, which further strengthen plant stress responses and regulate tolerance mechanisms. Understanding the TOR–SnRK pathway provides a deepened understanding of how plants regulate energy under toxic environmental conditions. In addition to these, targeting these pathways assists in designing crops and agricultural products that are more resilient to heavy metal toxicity, promoting sustainable agriculture in contaminated areas.

## Introduction

Heavy metal contamination resulting from industrialization and anthropogenic activities has emerged as a major environmental concern, severely affecting crop productivity, soil quality and ecosystem stability (Mishra et al., 2023; Kotnala et al., 2025). The heavy metals such as As, Cd, Hg, Cr, and Pb are non-biodegradable and highly toxic elements that can accumulate in plant systems leading to oxidative stress, impaired growth and metabolic disruption (Rahman et al., 2019; Rai et al., 2019; Naaz et al., 2023b; Bhat et al., 2025; Mohamed et al., 2025; Shetty et al., 2025).

At elevated concentrations, both essential and non-essential metals induce similar toxic effects in plants, like chlorosis, less nutrient uptake, stunted growth, reduced biomass, impaired photosynthesis, low development, and ultimately plant death (Naaz et al., 2023a).

To survive in heavy metal stress plant evolved diverse defence mechanisms and signaling networks which enable them to adapt to toxic environmental conditions (Oraby et al., 2025; Sharma et al., 2025). Under stress conditions, plants strategically reallocate resources by downregulating energy-intensive anabolic pathways, such as protein and nucleotide biosynthesis, while activating catabolic and protective processes that conserve energy and enhance stress adaptation (Xu and Fu, 2022). A central component of this adaptive response involves the balance between growth-promoting and stress-responsive pathways. The Target of Rapamycin (TOR) kinase acts as a master regulator of anabolic growth processes in response to nutrient and energy availability, whereas Sucrose Non-Fermenting-1-Related Protein Kinase (SnRKs) particularly, SnRK1 and SnRK2, function as key mediators of energy deprivation and abscisic acid (ABA)-dependent stress signaling (Jamsheer et al., 2019; Lim and Lee, 2023; Son and Park, 2023; Han et al., 2024; Liu et al., 2025). Antagonistic interactions between TOR and SnRK1 have been well characterized under nutrient limitation and other abiotic stresses, where TOR promotes growth and SnRK1 enhances survival-oriented metabolic reprogramming (Dobrenel et al., 2016; Jamsheer et al., 2019; Artins et al., 2024; Li et al., 2024; Rabeh et al., 2024; Feng et al., 2025; Yang et al., 2025).

Both TOR and SnRK signaling pathways constitute a central regulatory axis controlling the balance between anabolic growth and stress-induced catabolic reprogramming. Under energy-limited conditions, activation of SnRK1 promotes catabolic metabolism and autophagy-related gene expression, whereas TOR activity sustains biosynthetic processes and suppresses autophagy (Feng et al., 2025). Heavy metal exposure, particularly cadmium (Cd) and iron (Fe) toxicity, leads to reactive oxygen species (ROS) accumulation, redox imbalance, and metabolic disruption (Kulik et al., 2011; Dobrenel et al., 2016).

Such disturbances are reported to affect the antagonistic interaction between TOR and SnRK signaling pathways, particularly under conditions of nutrient deprivation and abiotic stress. Emerging evidence from research suggests that Cd and Pb activate SnRK-dependent antioxidant responses, while ABA-activated SnRK2 kinases may suppress TOR activity during stress adaptation. Additionally, arsenic (As) has been reported to inhibit TORC1 in other eukaryotic systems, indicating potential metal-specific modulation of TOR signaling (Hosiner et al., 2008; Ansari et al., 2009; Kulik et al., 2012; Wang et al., 2018; Zhao et al., 2023; Eom et al., 2024). Collectively, these findings support a model in which heavy metal-induced ROS and hormonal signaling may shift the TOR–SnRK balance toward survival-oriented metabolic programs, although direct mechanistic validation in plants under metal stress remains limited.

A number of studies have reported that TOR and SnRK pathways interact in a complex manner to regulate plant metabolic activities, however, their coordinated roles under heavy metal stress remain poorly understood. While the functions of TOR and SnRK signaling are well recognized in regulating plant growth, metabolism, and responses to various abiotic stresses (Feng et al., 2025), their specific regulatory interplay during heavy metal toxicity has not been comprehensively explored. Furthermore, the available evidence linking heavy metal-induced oxidative stress, hormonal signaling, and TOR–SnRK regulatory balance remains scattered across different studies and has not yet been synthesized in a unified conceptual framework. Therefore, this mini-review aims to integrate current knowledge on TOR and SnRK signaling pathways with emerging insights into heavy metal-induced stress responses in plants. Specifically, the review focuses on the potential mechanistic connections between TOR–SnRK signaling, ROS-mediated stress pathways, and metabolic reprogramming during heavy metal exposure, highlighting key knowledge gaps and future research directions that may help clarify how plants balance growth and survival under metal toxicity. Importantly, this review does not aim to provide a general overview of abiotic stress signaling but rather focuses on integrating emerging evidence of TOR–SnRK signaling with heavy metal-induced oxidative stress responses. By synthesizing dispersed findings and highlighting potential regulatory connections, this review proposes a conceptual framework for understanding how growth–stress signaling networks may regulate plant adaptation to heavy metal toxicity.

## Impact of heavy metals on plant physiology and metabolism

**Figure 1:**
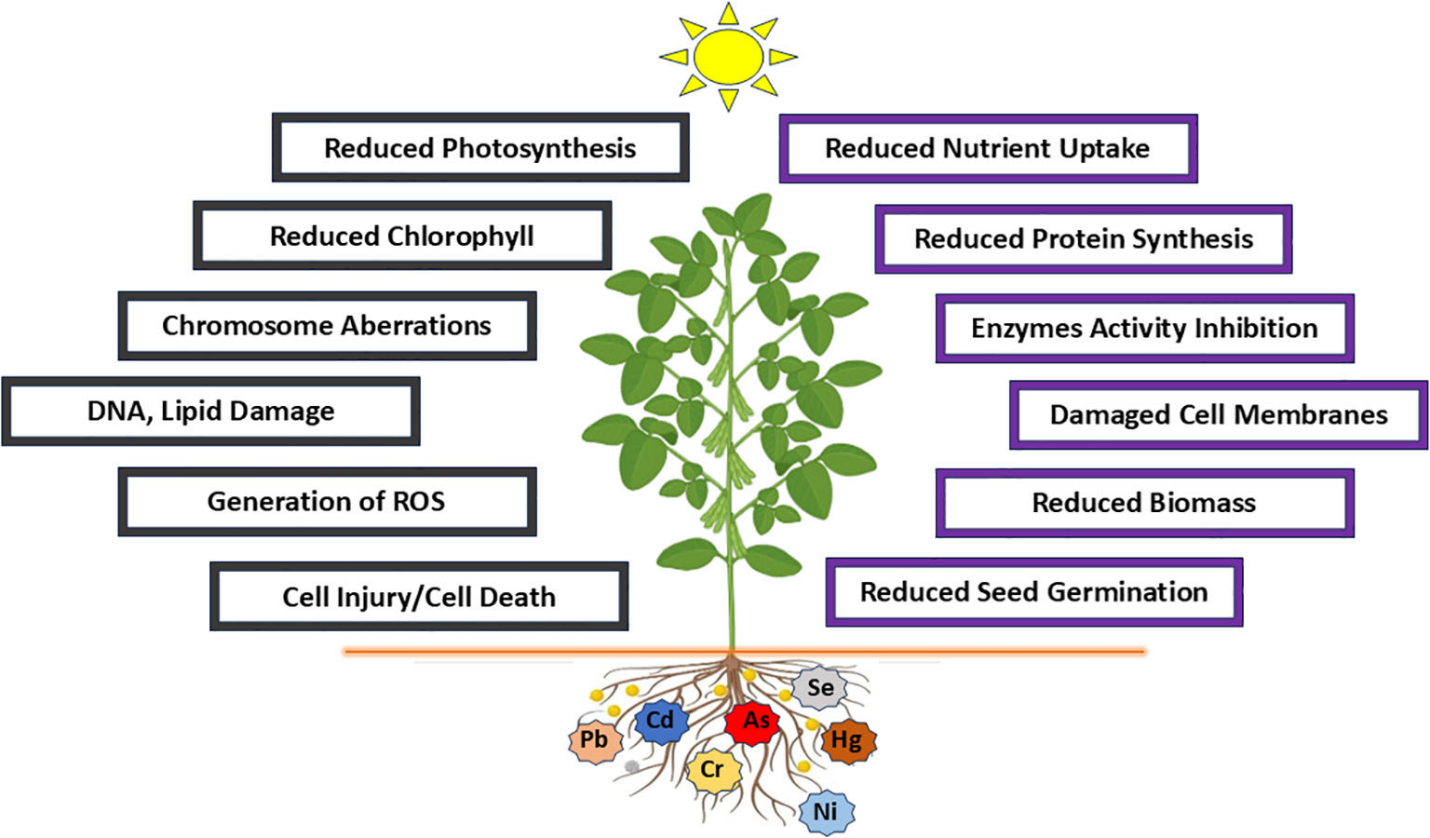
Effects of heavy metal stress on plant physiology and development. Heavy metals affect vital physiological processes such as photosynthesis, nutritional balance, growth, and antioxidant equilibrium, resulting in oxidative stress, metabolic changes, and reduced plant growth.

Heavy metals challenge plants through multiple, interconnected mechanisms, with oxidative stress being a primary consequence as a result of overproduction of ROS, which can disrupt lipids, proteins, and nucleic acids, impairing essential cellular processes (Berni et al., 2019; Gechev and Petrov, 2020; Mansoor et al., 2023; Huang et al., 2025). They also disrupt nutrient homeostasis; for example, cadmium (Cd) competes with vital elements like zinc (Zn), iron (Fe), and calcium (Ca) for transporters, causing deficiencies and metabolic imbalances (Rai et al., 2019; Naaz et al., 2023a). In addition, heavy metals—including Cd, Pb, Hg, and As can disrupt photosynthesis by damaging photosystems, reducing chlorophyll content, and inhibiting carbon fixation, thereby limiting the plant’s ability to generate energy (Angon et al., 2024; Vitelli et al., 2024; Umar et al., 2025). Beyond these direct effects, plants respond through metabolic reprogramming, redirecting carbon fluxes and energy from growth toward detoxification and defense pathways (Xu and Fu, 2022; Feng et al., 2025). The direct and indirect effects of heavy metal stress on plant physiology and development are listed in **Figure 1**.

Heavy metals further influence key signaling networks, including calcium fluxes, phytohormone regulation, and transcriptional control, which together coordinate adaptive stress responses (Parwez et al., 2023; Niekerk et al., 2024).

## Metal-specific uptake, transport, and compartmentation

Due to variations in metal absorption, transport, and intracellular sequestration systems, plants react differently to specific heavy metals. Specific or non-specific transporters allow toxic metals to enter plant cells, affecting their cellular distribution and main harmful locations (Clemens, 2006; Dalcorso et al., 2013). For example, arsenate (AsV) is transported via phosphate transporters because of its structural similarities to phosphate (Tripathi et al., 2012; Gautam et al., 2020), whereas cadmium (Cd) is frequently taken up via transporters for important divalent cations like zinc (Zn) and iron (Fe), including ZIP and NRAMP family proteins (Dalcorso et al., 2013). Lead (Pb) and mercury (Hg) can reduce their mobility but cause localized toxicity by interacting with membrane transport mechanisms or binding firmly to components of cell walls (Viehweger, 2014; Page and Feller, 2015).

Once inside the plant, ABC transporters and metal-chelating molecules like phytochelatins and metallothioneins work to selectively compartmentalize metals into vacuoles, cell walls, or organelles (Clemens and Ma, 2016). Reactive oxygen species (ROS) production, energy imbalance, and metabolic disturbance are all influenced by the degree of cytosolic metal buildup, which is determined by these activities.

Furthermore, the activation of TOR and SnRK signaling pathways is probably going to be impacted by these metal-specific variations in absorption and compartmentation (Baena-González, 2010). For instance, metals that primarily cause localized oxidative stress may variably alter ROS-dependent signaling cascades, while metals that significantly impair mitochondrial function and ATP synthesis may preferentially activate SnRK1-mediated energy stress signaling (Jamsheer et al., 2019). Therefore, a crucial approach for understanding how different heavy metals may differentially affect the TOR–SnRK signaling axis is provided by integrating metal-specific transport and sequestration mechanisms (Dobrenel et al., 2016).

These combined effects emphasize the need for flexible and efficient signaling networks that allow plants to adapt to heavy metal stress.

## Tissue and cell type specific aspects of TOR–SnRK regulation

The effects of heavy metal stress and TOR–SnRK signaling likely differ across various tissues and cell types based on where the metal accumulates and which physiological processes are affected (Dalcorso et al., 2013; Clemens and Ma, 2016). Roots are the primary site for metal uptake and initial stress detection. Here, the TOR–SnRK signaling may control nutrient transport, ion balance, detoxification, and root growth responses (Jamsheer et al., 2019; Liu et al., 2020; Parwez et al., 2023). In photosynthetic tissues, heavy metals can disrupt chloroplast function and carbon metabolism. This disruption might affect TOR-dependent growth regulation and SnRK-mediated energy stress signaling (Baena-González, 2010; Dobrenel et al., 2016). Additionally, guard cells and vascular tissues may help plants adapt to stress through ABA-dependent signaling, stomatal control, and long-distance transport processes (Szymańska et al., 2019). While direct evidence from specific tissues under heavy metal stress is still limited, these findings imply that TOR–SnRK signaling may work in a coordinated way to balance growth, defense, and metabolic adaptation when exposed to heavy metals (Margalha et al., 2019; Sharma et al., 2022).

## TOR signaling in the development and metabolism of plants

Target of Rapamycin (TOR) is a highly conserved Ser/Thr kinase that forms the TOR Complex 1 (TORC1) in plants, functioning as a key regulator of growth and development (Wullschleger et al., 2006). TOR plays a major role in coordinating growth-related processes and responses to environmental stresses (Liu et al., 2025). TOR integrates nutrient availability, energy status, and hormone inputs to stimulate anabolic activities such as protein synthesis, ribosome biogenesis, and nucleotide biosynthesis (Meng et al., 2022). Its activity is significantly controlled by upstream regulators such as auxin, glucose, and amino acids, which collectively boost TOR signaling under favourable growth conditions (Shi et al., 2018; Haq et al., 2022).

The plant TOR complex consists of three major components—TOR kinase, RAPTOR (Regulatory-Associated Protein of TOR), and LST8—which together regulate cell growth, protein synthesis, and metabolic activity (Rabeh et al., 2024). TOR activates downstream effectors such as S6 kinase (S6K) and other translational regulatory components, thereby promoting protein synthesis, mRNA translation, and biomass accumulation (Xiong et al., 2013; Dobrenel et al., 2016). Importantly, TOR acts as a negative regulator of autophagy when conditions are optimal. However, when there is an energy deficit or oxidative stress, TOR activity decreases. This change leads to less anabolic growth and triggers catabolic and stress-adaptive pathways, including autophagy. Feng et al. (2025) revealed that accumulation of ROS and energy-deficit conditions suppresses TOR-S6K signalling and activates autophagy. Heavy metal stress causes excessive ROS production, energy deprivation, and mitochondrial disruptions in plants, suggesting that toxic metals inhibit TOR activity in some way through these upstream signals (Pu et al., 2017; Rabeh et al., 2024; Feng et al., 2025). Cellular resources such as antioxidant defense, detoxification pathways are redirected from development to plant survival during stress when TOR activity is suppressed in plants (Wullschleger et al., 2006; Dobrenel et al., 2016; Haq et al., 2022; Rabeh et al., 2024). Although the role of TOR is well characterized in stress responses, there are only a few studies that have examined the role of TOR in heavy metal stress, and they are restricted to rice and Arabidopsis (Li and Zhao, 2024; Li et al., 2024).

Further evidence reported that in conditions of oxidative stress and energy limitation generated by heavy metals, TOR signaling is blocked, resulting in the downregulation of anabolic activities and the redirection of cellular resources toward protection and detoxification (Tiwari and Lata, 2018). In a study, Xiong et al. (2013) showed that glucose activates the TOR pathway, coordinating metabolism and growth. They observed that oxidative stress, which is frequently produced by environmental contaminants, suppresses TOR signaling by lowering the phosphorylation of critical downstream effectors such as S6 kinase and eIF4E-binding proteins (Tiwari and Lata, 2018; Fu et al., 2020). These findings revealed that under stressful conditions of heavy metal toxicity, energy and nutrient imbalances suppress TOR activity as TOR is a significant regulator integrating nutrient, energy, and stress signals, and its downregulation in the presence of heavy metal stress might be essential for enabling plants to activate defense pathways, and adapt to unfavourable environmental conditions.

## SnRK signaling: integrating energy and stress responses

Sucrose non-fermenting-1-related kinases (SnRKs) are a large family of kinases that control energy and stress signals in plants (Hasan et al., 2022). They are divided into three major subfamilies: SnRK1, SnRK2, and SnRK3 (Mondal et al., 2024). Genome-wide analyses indicate that SnRK genes have undergone several duplication events, resulting in functional diversification across SnRK1, SnRK2, and SnRK3 subfamilies, each with distinct functions in stress adaptation (Zhu et al., 2020). Promoter analyses reveal ABA-related elements and stress-responsive miRNA sites, suggesting evolutionary adaptation of SnRKs for precisely regulated stress responses, including their potential roles in heavy metal tolerance (Mondal et al., 2024; Zheng et al., 2025).

SnRK1 is a heterotrimeric serine-threonine protein kinase complex comprising two regulatory subunits, β and γ, and an α catalytic subunit. The SnRK1s can be categorized into three subclasses: SnRK1.1, SnRK1.2, and SnRK1.3, with the α subunit encoded by *SnRK1α1*, *SnRK1α2*, and *SnRK1α3* (*KIN10/KIN11/KIN12*) in Arabidopsis, and most SnRK1 activity is attributed to *SnRK1α1* (Crozet et al., 2014). SnRK1, which is related to yeast SNF1 and mammalian AMPK, functions as a metabolic sensor activated under low-energy or stressed conditions. Its activation promotes catabolic pathways while repressing energy-demanding anabolic processes, thereby conserving cellular energy (Han et al., 2024). SnRK1 promotes autophagy, enabling the removal of damaged proteins and organelles, and induces transcription of stress-responsive genes that mitigate toxicity (Soto-Burgos and Bassham, 2017; Haq et al., 2022). It also antagonizes TOR activity, thereby reinforcing energy conservation and redirecting resources toward defense (Soto-Burgos and Bassham, 2017). Through these coordinated actions, SnRK pathways act as crucial regulators that help plants to survive in heavy-metal contaminated areas (Baena-González and Sheen, 2008; Feng et al., 2022; Mondal et al., 2024).

SnRK2 kinases are predominately involved in abscisic acid (ABA)-mediated responses to drought, salinity, and osmotic stress (Yang et al., 2019). Under heavy metal exposure, SnRK2 is rapidly activated and serves a crucial role in enhancing stress tolerance. Direct evidence of SnRK2 activation under Cd, Pb, and As exposure has been demonstrated in Arabidopsis and rice (Kulik et al., 2011, 2012; Gu et al., 2021; Zhao et al., 2023).

Kulik et al. (2012) confirmed that SnRK2 kinases, particularly *SnRK2.4*, are transiently activated under Cd stress. A study showed that *SnRK2.4* knockout mutants had better root growth tolerance to Cd compared to wild-type plants, along with lower accumulation of ROS. This highlights SnRKs as key regulators in a plant’s response to heavy metal stress. Their data strongly suggest that SnRK2 plays a role in heavy metal tolerance. A recent study showed that ABA-induced SnRK2 activation increases the binding of Cd to root cell walls, reduces its translocation to shoots, and upregulates protective antioxidants, thereby associating SnRK activation with heavy-metal detoxification (Zhao et al., 2023). Notably, other SnRK2 kinases independent of ABA,  such as *SnRK2.4* and *SnRK2.10*, are also engaged in the regulation of ROS and root growth under Cd and osmotic stress. These results show that SnRK2 could mediate a combination of ABA-dependent and ABA-independent signals in metal stress tolerance (Szymańska et al., 2019; Hasan et al., 2022). A study by Xu et al. (2022) identified that *SnRK2.7* expression is upregulated under Cd stress, which enhances Cd tolerance, confirming the role of SnRK2s in stress signaling pathways in *Paspalum vaginatum* Sw.

SnRK3 (CIPKs) interact with calcineurin B-like proteins (CBLs) to mediate calcium-dependent stress signaling (CBL–CIPK) (Luan, 2009; Tang et al., 2020). Under heavy metal exposure, SnRK3 maintains ion homeostasis, regulates ROS, and modulates root growth, connecting calcium signaling with metabolic and stress adaptation networks (Szymańska et al., 2019; Ma et al., 2020; Mondal et al., 2024).

Through these coordinated actions, SnRK pathways act as crucial mediators of plant survival in environments exposed to heavy metal toxicity. Together, SnRK1, SnRK2, and SnRK3 act as a central regulator coordinating energy status, redox balance, and stress adaptation. Their complementary and sometimes antagonistic actions with TOR allow plants to prioritize survival, detoxification, and limited growth under heavy metal stress (Baena-González, 2010; Mondal et al., 2024; Feng et al., 2025).

## TOR–SnRK crosstalk in heavy metal stress

The relationship between TOR and SnRK signaling is characterized by antagonism yet intricate interconnection, allowing plants to coordinate between growth and survival. SnRK1 and TOR act as central cellular energy sensors that coordinate metabolic reprogramming under stress conditions. While TOR promotes anabolic growth processes under nutrient sufficiency, SnRK1 becomes activated during energy deprivation and redirects metabolism toward catabolic pathways such as autophagy and nutrient recycling (Feng et al., 2025). Different heavy metals may influence TOR–SnRK signaling through distinct cellular pathways. For instance, cadmium (Cd) toxicity primarily triggers oxidative stress and energy depletion, leading to activation of SnRK kinases and repression of TOR-mediated growth signaling. Studies in Arabidopsis have shown that Cd exposure induces transient activation of SnRK2 kinases, indicating the involvement of stress-responsive signaling pathways in TOR–SnRK regulation (Kulik et al., 2012). In contrast, arsenic (As) has been reported to inhibit TORC1 activity in other eukaryotic systems, suggesting that certain metals may directly suppress TOR signaling independently of classical energy-stress pathways (Hosiner et al., 2008; Ansari et al., 2009). Lead (Pb) toxicity also disrupts photosynthesis and redox homeostasis, which may indirectly influence TOR–SnRK signaling by altering cellular metabolic status (Zhao et al., 2026). These observations suggest that different metals activate distinct upstream stress signals that ultimately converge on the TOR–SnRK regulatory network. Under Cd exposure, ROS accumulation and ATP depletion are likely to influence *SnRK1.1* mediated energy stress signaling, which may promote phosphorylation of RAPTOR (Regulatory-Associated Protein of TOR), a regulatory component of TORC1 that recruits substrates to the TOR kinase complex. RAPTOR phosphorylation suppresses TOR activity. Reduced TOR activity may in turn relieve TOR-mediated inhibition of autophagy, thereby contributing to metabolic recycling and stress adaptation. This proposed mechanism is supported by studies demonstrating SnRK1–TOR antagonism and RAPTOR regulation under broader abiotic stress and energy stress conditions, although direct molecular evidence under plant heavy metal stress remains limited (Li and Zhao, 2024; Zhao et al., 2026).

FCS-like zinc finger (FLZ) proteins, a distinct class of C2-C2-type zinc finger proteins discovered only in terrestrial plants, connect the TOR and SnRK1 pathways (Feng et al., 2025). Recent studies have identified FLZ proteins as plant-specific regulatory adaptors that integrate SnRK1 and TOR signaling. FLZ proteins physically interact with the catalytic subunits of the SnRK1 complex and modulate its activation through regulation of T-loop phosphorylation. Several FLZ proteins also interact with components of the TOR complex, thereby acting as molecular scaffolds that coordinate antagonistic SnRK1–TOR signaling under stress conditions (Jamsheer et al., 2019). Recent studies further revealed that some FLZ proteins interact with autophagy-related proteins such as ATG8, linking energy-sensing signaling pathways with autophagy regulation and stress adaptation in plants (Jamsheer et al., 2018). During low energy levels and upon activation of SnRK1, FLZs may facilitate the recruitment of SnRK1 to certain cellular compartments and substrates, enabling it to phosphorylate critical regulatory proteins that initiate autophagy (Yang et al., 2025).

FLZ binding to TOR subunits may serve to balance the contrary SnRK1 and TOR signaling pathways, resulting in a dynamic and coordinated response to plant energy status. FLZs integrate various stress and metabolic signals by connecting the SnRK1 and TOR complexes (Artins and Fernie, 2023), allowing plants to rapidly adjust their physiology and resource allocation in response to changing environmental conditions by precisely modulating autophagy and other metabolic pathways. In addition to RAPTOR-mediated TOR inhibition, SnRK1 can directly regulate autophagy by phosphorylating key autophagy-related proteins such as ATG1, thereby promoting autophagosome formation and cellular recycling during energy stress (Jamsheer et al., 2018).

Under heavy metal-induced energy stress, FLZ-mediated modulation may help maintain a balance between catabolic stress responses and residual TOR-driven growth signaling (Mitra et al., 2023). Inhibition of TOR is likely to suppress energetically intensive processes such as ribosome biogenesis and protein synthesis, thereby conserving valuable resources under stress conditions (Gui et al., 2023). SnRK1 further reprograms metabolism toward ATP conservation and the synthesis of defense-related metabolites, potentially enhancing stress tolerance (Han et al., 2024). Despite TOR suppression, partial TOR activity may be retained in certain contexts to support essential cellular repair and limited protein synthesis required for recovery. Collectively, these interactions are proposed as a working model for TOR–SnRK crosstalk under heavy metal stress, potentially contributing to the balance between stress adaptation and growth recovery under favourable conditions.

In addition to RAPTOR and FLZ proteins, several upstream regulatory signals contribute to the modulation of TOR–SnRK crosstalk under stress conditions. Cellular energy status, reflected by changes in the ATP/AMP ratio, is a key determinant of SnRK1 activation, enabling plants to rapidly shift metabolic priorities during stress (Zhao et al., 2026). Hormonal signaling pathways, particularly abscisic acid (ABA), also participate in this regulation through activation of SnRK2 kinases, which can antagonize TOR activity during stress responses (Baena-González and Sheen, 2008; Dobrenel et al., 2016; Jamsheer et al., 2019; Eom et al., 2024). Furthermore, stress-responsive kinases such as General Control Nonderepressible 2 (GCN2) integrate nutrient and translational control with TOR–SnRK signaling, collectively coordinating metabolic reprogramming and stress adaptation (Halford and Hey, 2009; Feng et al., 2025).

Kulik et al. (2012) demonstrated that SnRK2 kinases are transiently activated under Cd stress, highlighting a potential mechanism by which SnRK2 modulates growth and stress responses, possibly through antagonism with TOR signaling. Modulation of TOR or SnRK1 genes significantly impacts abiotic stress tolerance. For instance, overexpressing *SnRK1α1* in Arabidopsis improves drought resistance, while decreasing SnRK1 levels increases stress sensitivity to abiotic stress (Jamsheer et al., 2019; Rodriguez et al., 2019), highlighting the antagonistic relationship between SnRK1 and TOR. Although these studies focus on other abiotic stresses, they provide an important mechanistic framework for understanding how SnRK1-mediated metabolic reprogramming may contribute to heavy metal stress tolerance.

SnRK1, located upstream of TOR, regulates autophagy in response to salt, osmotic, and nutritional stresses, suggesting that it may also play a role in heavy metal detoxification by reducing metal-induced cytotoxicity and protecting energy balance (Soto-Burgos and Bassham, 2017; Haq et al., 2022).

During mild stress, TOR promotes anabolic processes and cell proliferation by activating downstream effectors including S6K and 4E-BP-like proteins, whereas SnRK1 and SnRK2 function as metabolic checkpoints that reprogram transcriptional and metabolic networks under severe metal toxicity (Feng et al., 2025). SnRKs are activated, thereby inhibiting TOR signaling and leading to the induction of autophagy and reallocation of energy toward defense and detoxification. This antagonistic crosstalk ultimately determines if a plant cell would prefer survival strategies or suppressed growth in response to heavy metal stress (Rodriguez et al., 2019; Eom et al., 2024; Feng et al., 2025).

**Figure 2:**
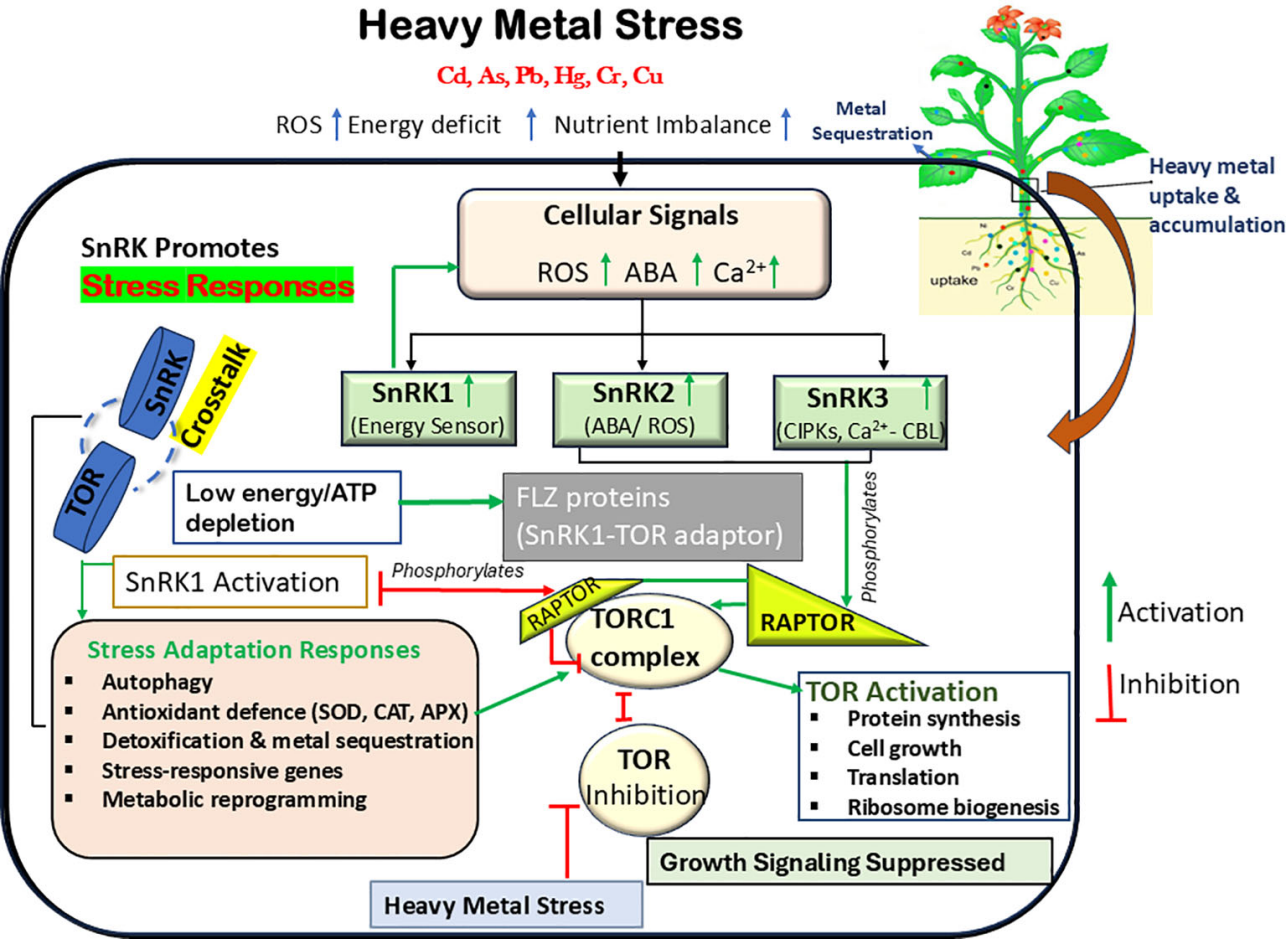
The proposed model depicts the crosstalk between the TOR and SnRK signaling pathways under heavy metal stress in plants. Heavy metal stress (Cd, As, Pb, Hg, Cr, Cu, Zn, Ni) causes metal uptake and transport from roots to aerial tissues, leading to ROS production, ABA signaling, Ca^2+^ flux, and cellular toxicity. These signals activate SnRK kinases (SnRK1, SnRK2, and SnRK3), which promote defense responses such as autophagy, antioxidant activity, metal detoxification, and the expression of stress-responsive genes. Activated SnRK1 promotes stress adaptation responses and inhibits TORC1 signaling through RAPTOR phosphorylation and FLZ adaptor proteins, resulting in suppression of growth-related processes and enhancement of stress tolerance mechanisms. Mechanistically, heavy metal stress triggers ROS accumulation and ATP depletion, leading to SnRK1 activation, RAPTOR phosphorylation, TOR inhibition, and subsequent induction of autophagy and metabolic recycling.

Eom et al. (2024) also confirmed that during stress, TOR activity is modulated in coordination with SnRK1 to maintain energy balance and promote stress-adaptive responses. Collectively, these findings support a mechanistic model in which heavy metal–induced oxidative and energy stress activates SnRK signaling, which in turn suppresses TOR activity through regulators such as RAPTOR and FLZ proteins, thereby promoting autophagy, metabolic reprogramming, and stress adaptation while maintaining the capacity for growth recovery when favourable conditions return. A proposed model that depicts the crosstalk between TOR and SnRK signalling under heavy metal stress is provided in **Figure 2**.

## Integration of signalling networks with hormonal, redox, and calcium signaling

Heavy metal stress triggers a coordinated signaling network involving ROS production, calcium influx, and hormonal signaling that converges on the TOR–SnRK regulatory module. Early stress perception often induces rapid ROS accumulation and transient Ca^2+^ fluxes, which act as primary secondary messengers. These signals stimulate ABA biosynthesis and activate ABA-dependent kinases such as SnRK2s. Increased ABA and ROS signaling together promote activation of SnRK pathways while suppressing TOR activity, thereby shifting cellular priorities from growth toward stress defense. Through this coordinated signaling network, plants integrate cellular metabolic status with environmental stress cues to regulate antioxidant defenses, ion homeostasis, and detoxification mechanisms under heavy metal stress. The management of plant responses in heavy metal contaminated environments is carefully regulated through hormonal, redox, and calcium signaling networks that interact with TOR and SnRK pathways (Mitra et al., 2023). Hormones such as ABA, ethylene, and salicylic acid synergize with SnRKs to amplify stress-responsive mechanisms, whereas auxin has been shown to encourage TOR signaling, particularly during growth recovery following stress alleviation (Ghori et al., 2019). Species-specific differences in ABA-mediated TOR–SnRK regulation have also been reported. ABA-induced SnRK2 activation and TOR suppression show stronger effects in Arabidopsis and rice than in some legume species (Hasan et al., 2022; Eom et al., 2024). Zhao et al. (2023) showed that ABA enhances Cd binding to root cell walls, limits Cd translocation to shoots, and boosts antioxidant defense. These protective effects occur through ABA-induced activation of SnRK2 kinases, which regulate stress-responsive genes. In the presence of heavy metal-induced oxidative stress, ABA-mediated signaling regulates stomatal action via ROS production and *SnRK2.*6/*OST1*–mediated activation, thus enhancing stress adaptation responses like stomatal closure. These processes could likely interact with TOR–SnRK signaling networks that orchestrate energy homeostasis, ROS signaling and growth regulation in response to stress (Ma et al., 2020; Yu et al., 2023).

Redox regulation represents a key layer of integration, as ROS function both as damaging molecules and as secondary messengers in stress signaling. SnRK1 activation enhances antioxidant gene expression and induces autophagy, thereby maintaining redox balance under heavy metal toxicity (Yang et al., 2023). H_2_ S modulates antioxidant defenses by increasing antioxidant enzyme activities, maintaining ion homeostasis, and mitigating ROS-mediated oxidative damage (Munteanu et al., 2023). Although the direct connection between H_2_ S and the TOR–SnRK signaling network remains underexplored, H_2_ S-mediated modulation of redox and energy homeostasis suggests a potential influence on these stress-sensing kinases (Zhao et al., 2022). ROS signaling also interacts closely with calcium signaling pathways, where ROS-induced Ca^2+^ influx further amplifies ABA signaling and downstream SnRK activation.

Calcium signaling acts as a rapid and versatile mediator of stress perception. Heavy metal-induced Ca^2+^ fluxes activate CBL–CIPK complexes (SnRK3), linking ion homeostasis with stress adaptation mechanisms (Shi et al., 2023). MAPK pathways interact with ROS and ABA signaling to modulate stress adaptation mechanisms (Manna et al., 2023). Collectively, these interactions reveal the intricate network of TOR–SnRK signaling within plant regulatory systems, enabling plants to respond effectively and appropriately to different heavy metal stresses (Gechev and Petrov, 2020; Jiang et al., 2022; Saile et al., 2023). Heavy metal exposure initiates a coordinated signaling cascade in which ROS production, Ca^2+^ influx, and ABA signaling operate as interconnected early stress signals. Metal-induced oxidative stress stimulates ROS accumulation, which activates Ca^2+^ permeable channels in the plasma membrane and promotes cytosolic Ca^2+^ transients. These Ca^2+^ signals are sensed by CBL–CIPK complexes and calcium-dependent protein kinases, which further amplify ROS signaling and stimulate ABA biosynthesis. ABA signaling then activates SnRK2 kinases and promotes SnRK1-mediated energy stress signaling, while simultaneously suppressing TOR activity to restrict growth-related anabolic processes. Through this interconnected signaling cascade, ROS, Ca^2+^, and ABA collectively function as upstream regulators that modulate the antagonistic TOR–SnRK signaling module, enabling plants to rapidly redirect cellular metabolism from growth toward stress defense, detoxification, and survival under heavy metal stress. The integration of ABA signaling with ROS generation and Ca^2+^ flux ultimately converges on the TOR–SnRK regulatory module. This coordinated signaling network promotes SnRK activation and TOR inhibition, enabling plants to shift from growth-related metabolism to defense-oriented processes such as antioxidant activation, ion sequestration, and detoxification under heavy metal stress (Keyster et al., 2020; Liu et al., 2020; Zhao et al., 2023; Feng et al., 2025). Thus, the TOR–SnRK regulatory module functions as a central integrative hub that translates hormonal, redox, and calcium signals into coordinated metabolic and transcriptional responses, enabling plants to balance growth and survival under heavy metal stress conditions.

## Biotechnological applications: targeting TOR–SnRK pathways

The development of crop cultivars with improved resistance to heavy metals is made possible by advancing our understanding of TOR–SnRK interactions. Scientific findings revealed that genetic engineering techniques, like SnRK1 overexpression, enhance stress resilience by promoting metabolic adaptability, encouraging autophagy, and strengthening antioxidant defenses. Overexpression of SnRK1α enhanced salt tolerance in tomato by improving ROS detoxification and ABA signaling (Morales-Herrera et al., 2024; Xu et al., 2025), indicating a broader role in stress adaptation. Similarly, expression of the wheat *TaSnRK2.8* gene increased drought, salt, and cold tolerance in Arabidopsis (Zhang et al., 2010). Studies in Arabidopsis and rice further demonstrate that SnRK1 regulates stress-responsive gene expression and metabolic reprogramming during environmental stress (Cho et al., 2012), while genome-wide analyses in wheat highlight the role of SnRK genes in crop stress resilience (Mishra et al., 2023). These findings suggest that manipulation of energy-sensing kinases can enhance stress tolerance mechanisms relevant to heavy metal exposure. Partial inhibition of TOR activity has been shown to enhance stress tolerance while barely impacting growth, making it an effective technique for maintaining a balance between productivity and resilience (Wullschleger et al., 2006; Jamsheer K et al., 2022; Morales-Herrera et al., 2024; Xu et al., 2025). Experimental studies also highlight the role of TOR signaling in stress adaptation. Chemical inhibition of TOR in *Arabidopsis thaliana* activated autophagy and enhanced stress tolerance under nutrient-limiting conditions (Dobrenel et al., 2016), suggesting a conserved mechanism that may also operate under heavy metal stress. In addition, ABA-activated SnRK2 kinases were shown to suppress TOR activity, shifting cellular metabolism from growth to stress defense (Shu et al., 2026). Modern gene-editing technologies such as CRISPR–Cas9 provide precise tools to modify components of the TOR–SnRK signaling network. For example, targeted editing of regulatory genes involved in energy and stress signaling in crops such as rice and tomato has demonstrated improved tolerance to abiotic stresses by altering metabolic and antioxidant pathways (Kaur et al., 2025), which may have implications for heavy metal stress tolerance.

Nanoparticles emerged as an effective approach for enhancement of plant abiotic stress tolerance. They enhance plant resistance to heavy metal toxicity by upregulating antioxidant defences and sustaining metabolic balance (Al-Khayri et al., 2023). TOR and SnRK signaling also mediate these responses, and studying these interactions could reveal new pathways involved in heavy metal tolerance (Zhou et al., 2020; Sen et al., 2025). Additionally, TOR inhibitors and potential SnRK activators offer promising opportunities to enhance tolerance via chemical modification (Khan et al., 2021; Parwez et al., 2023; Ni et al., 2025). Collectively, these strategies highlight the importance of understanding the TOR–SnRK signaling for crop improvement plans promoting sustainable agriculture in toxic metal-contaminated areas. Despite these promising advances, manipulation of the TOR–SnRK signaling network presents several challenges. Because these kinases regulate fundamental processes such as growth, metabolism, and energy homeostasis, excessive TOR inhibition or constitutive SnRK activation may negatively affect plant growth and yield. Additionally, the complexity and cross-talk of these pathways with hormonal and redox signaling may lead to unintended physiological effects. Therefore, precise spatial and temporal regulation of these signaling components will be essential to ensure that improved stress tolerance does not compromise crop productivity. A summary of major factors and signalling pathways that influence plant adaptation to heavy metal stress are presented in **Table 1**. While several components of TOR–SnRK signaling have been directly investigated under heavy metal stress, some mechanisms summarized here are derived from studies on other abiotic stresses, such as drought, salinity, and cold. These studies provide a broader conceptual framework for understanding potential regulatory mechanisms under heavy metal stress.

**Table 1:** The table highlights the mechanisms, physiological effects on plants, and representative references for heavy metals (Cd, Pb, Hg, As), TOR, SnRK1/2/3 signaling modules, TOR–SnRK crosstalk, and their biotechnological applications..

Component	Key genes/ proteins	Plant species	Mechanism/role	Experimental evidence	Effect on plant	References
Heavy Metals Stress Signals (Cd, Pb, Hg, As)	RBOH antioxidant enzymes (SOD, CAT, APX)	Arabidopsis, rice, wheat	Induce ROS production, nutrient disruption, photosystem damage	Physiological assays, ROS measurements, gene expression analysis (Direct – heavy metal)	Chlorosis, stunted growth, metabolic reprogramming, oxidative damage	Gechev and Petrov, 2020; Rai et al., 2019; Riaz et al., 2021; Naaz et al., 2023a, 2023b; Bhat et al., 2025; Mohamed et al., 2025
TOR	TOR RAPTOR S6K	Arabidopsis, rice	Promotes growth & protein synthesis, inhibits autophagy, downregulates in heavy metal stress	Mutant analysis, TOR inhibitor studies, phosphorylation assays (Mixed evidence)	Supports growth under normal conditions, suppresses in heavy metal stress, facilitating defense and autophagy	Dobrenel et al., 2016; Li and Zhao, 2024; Li et al., 2024; Zhao et al., 2026; Sharma et al., 2022; Rohde et al., 2001; Jamsheer et al., 2019; Fu et al., 2020; Mitra et al., 2023; Zhao et al., 2023; Feng et al., 2025; Yang et al., 2022
SnRK1	*SnRK1α1* (*KIN10*) *SnRK1α2* (*KIN11*)	Arabidopsis, tomato	Acts as an energy/stress sensor, activates autophagy, inhibits TOR signaling, Enhances antioxidant defense and ABA-mediated responses under salt stress	Overexpression and mutant studies, transcriptional analysis, physiological stress assays (Inferred – abiotic stress)	Enhances stress tolerance, maintains redox balance, activates defense genes in heavy metal stress	Baena-González, 2010; Soto-Burgos and Bassham, 2017; Hasan et al., 2022; Peixoto and Baena-González, 2022; Yang et al., 2023; Mondal et al., 2024; Feng et al., 2025; Morales-Herrera et al., 2024; Xu et al., 2025
SnRK2	*SnRK2.4* *SnRK2.6* (*OST1*) *SnRK2.8*	Arabidopsis, rice, wheat	ABA-mediated stress signaling, ABA-dependent kinase that regulates stress-responsive gene expression and antioxidant defense	Knockout mutant analysis; kinase activity assays, Transgenic overexpression and physiological stress tolerance assays (Inferred – abiotic stress)	Regulates ROS, stress gene expression, root development under heavy metal stress, Improved tolerance to drought, salinity, and cold stress through enhanced ROS scavenging and stress signaling	Kulik et al., 2012; Szymańska et al., 2019; Zhao et al., 2023; Zhang et al., 2010; Cho et al., 2012; Mishra et al., 2023
SnRK3	CBL–CIPK complexes	Arabidopsis, rice	Ca^2+^-dependent signaling (CBL–CIPK)	Protein interaction assays; gene expression studies (Inferred – abiotic stress)	Maintains ion homeostasis, root growth, and stress adaptation under heavy metal exposure	Liu et al., 2020; Ma et al., 2020; Gu et al., 2021; Mondal et al., 2024
TOR–SnRK Crosstalk	SnRK1 RAPTOR FLZ proteins SnRK2 kinases TOR	Arabidopsis	SnRK1 inhibits TOR, promoting autophagy & defense activation, balances growth vs stress	Protein interaction and phosphorylation studies (Inferred – abiotic stress)	Optimizes growth vs defense under heavy metal stress; metabolic reprogramming and ROS detoxification	Dobrenel et al., 2016; Jamsheer et al., 2019; Margalha et al., 2019; Rodriguez et al., 2019; Belkov et al., 2020; Eom et al., 2024; Li and Zhao, 2024; Feng et al., 2025; Shu et al., 2026
Applications/Future perspective	SnRK1 TOR regulators	Arabidopsis, rice, tomato	Genetic engineering SnRK1 overexpression, TOR modulation, nanoparticles, and CRISPR-based editing	Transgenic studies, gene-editing experiments, physiological stress assays (Mixed evidence)	Improved heavy metal tolerance & crop resilience	Wullschleger et al., 2006; Khan et al., 2021; Sharma et al., 2022; Al-Khayri et al., 2023; Parwez et al., 2023; Morales-Herrera et al., 2024; Kaur et al., 2025; Xu et al., 2025

Direct – heavy metal, evidence derived from heavy metal stress studies; Inferred – abiotic stress, evidence derived from other abiotic stress conditions; Mixed evidence, combination of both.

## Concluding remarks and future prospects

Despite growing evidence supporting the regulatory role of TOR and SnRK pathways in abiotic stress, direct molecular evidence of their interaction under heavy metal stress remains limited. A significant gap exists in understanding the function of intermediate regulators, i.e., RAPTOR, FLZ-domain, and post-translational modifications like phosphorylation or ubiquitination. Before modulating these pathways, potential consequences should be considered, such as effects on crop development, nutrition imbalance, or stress sensitivity. Future research should combine hormonal, redox, and calcium signaling networks to better understand the specific molecular mechanisms underlying TOR–SnRK crosstalk under different heavy metal stress conditions. To determine the practical application of TOR–SnRK modification, field validation in crop species is also needed.

## References

[B1] Al-Khayri J. M. Rashmi R. Surya Ulhas R. Sudheer W. N. Banadka A. Nagella P. . (2023). The role of nanoparticles in response of plants to abiotic stress at physiological, biochemical, and molecular levels. Plants 12, 292. doi: 10.3390/PLANTS12020292. PMID: 36679005 PMC9865530

[B2] Angon P. B. Islam M. S. KC S. Das A. Anjum N. Poudel A. . (2024). Sources, effects and present perspectives of heavy metals contamination: Soil, plants and human food chain. Heliyon 10, e28357. doi: 10.1016/J.HELIYON.2024.E28357. PMID: 38590838 PMC10999863

[B3] Ansari M. K. A. Ahmad A. Umar S. Iqbal M. (2009). Mercury-induced changes in growth variables and antioxidative enzyme activities in Indian mustard. J. Plant Interact. 4, 131–136. doi: 10.1080/17429140802716713. PMID: 37339054

[B4] Artins A. Fernie A. R. (2023). FCS-like zinc finger proteins maintain energy homeostasis during stresses. Trends Plant Sci. 28, 1347–1349. doi: 10.1016/j.tplants.2023.09.004. PMID: 37743166

[B5] Artins A. Martins M. C. M. Meyer C. Fernie A. R. Caldana C. (2024). Sensing and regulation of C and N metabolism – novel features and mechanisms of the TOR and SnRK1 signaling pathways. Plant J. 118, 1268–1280. doi: 10.1111/TPJ.16684. PMID: 38349940

[B6] Baena-González E. (2010). Energy signaling in the regulation of gene expression during stress. Mol. Plant 3, 300–313. doi: 10.1093/MP/SSP113. PMID: 20080814

[B7] Baena-González E. Sheen J. (2008). Convergent energy and stress signaling. Trends Plant Sci. 13, 474–482. doi: 10.1016/J.TPLANTS.2008.06.006. PMID: 18701338 PMC3075853

[B8] Belkov V. I. И. Б.В. Garnik E. Y. Ю. Г.Е. Tarasenko V. I. И. Т.В. . (2020). Sugar-mediated regulation and the role of HXK1, SnRK1, TOR kinases in Arabidopsis thaliana. Izvestiya Vuzov. Prikladnaya Khimiya i Biotekhnologiya 10, 627–638. doi: 10.21285/2227-2925-2020-10-4-627-638

[B9] Berni R. Luyckx M. Xu X. Legay S. Sergeant K. Hausman J. F. . (2019). Reactive oxygen species and heavy metal stress in plants: Impact on the cell wall and secondary metabolism. Environ. Exp. Bot. 161, 98–106. doi: 10.1016/J.ENVEXPBOT.2018.10.017. PMID: 38826717

[B10] Bhat B. A. Rather M. A. Bilal T. Nazir R. Qadir R. U. Mir R. A. (2025). Plant hyperaccumulators: a state-of-the-art review on mechanism of heavy metal transport and sequestration. Front. Plant Sci. 16, 1631378. doi: 10.3389/FPLS.2025.1631378. PMID: 40772044 PMC12325257

[B11] Cho Y. H. Hong J. W. Kim E. C. Yoo S. D. (2012). Regulatory functions of SnRK1 in stress-responsive gene expression and in plant growth and development. Plant Physiol. 158, 1955. doi: 10.1104/pp.111.189829. PMID: 22232383 PMC3320198

[B12] Clemens S. (2006). Toxic metal accumulation, responses to exposure and mechanisms of tolerance in plants. Biochimie 88, 1707–1719. doi: 10.1016/j.biochi.2006.07.003. PMID: 16914250

[B13] Clemens S. Ma J. F. (2016). Toxic heavy metal and metalloid accumulation in crop plants and foods. Annu. Rev. Plant Biol. 67, 489–512. doi: 10.1146/ANNUREV-ARPLANT-043015-112301. PMID: 27128467

[B14] Crozet P. Margalha L. Confraria A. Rodrigues A. Martinho C. Adamo M. . (2014). Mechanisms of regulation of SNF1/AMPK/SnRK1 protein kinases. Front. Plant Sci. 5, 83320. doi: 10.3389/FPLS.2014.00190. PMID: 24904600 PMC4033248

[B15] Dalcorso G. Manara A. Furini A. (2013). An overview of heavy metal challenge in plants: from roots to shoots. Metallomics 5, 1117–1132. doi: 10.1039/C3MT00038A. PMID: 23739766

[B16] Dobrenel T. Caldana C. Hanson J. Robaglia C. Vincentz M. Veit B. . (2016). TOR signaling and nutrient sensing. Annu. Rev. Plant Biol. 67, 261–285. doi: 10.1146/annurev-arplant-043014-114648. PMID: 26905651

[B17] Eom S. H. Kim E. Hyun T. K. (2024). HXK, SnRK1, and TOR signaling in plants: Unraveling mechanisms of stress response and secondary metabolism. Sci. Prog. 107. doi: 10.1177/00368504241301533. PMID: 39636031 PMC11622374

[B18] Feng L. Li X. Zheng X. A. Zheng Z. Liu Q. R. Liu C. . (2025). SnRK1 and TOR: central regulators of autophagy in plant energy stress responses. aBIOTECH, 1–17. doi: 10.1007/S42994-025-00218-3. PMID: 41312094 PMC12647502

[B19] Feng X. Meng Q. Zeng J. Yu Q. Xu D. Dai X. . (2022). Genome-wide identification of sucrose non-fermenting-1-related protein kinase genes in maize and their responses to abiotic stresses. Front. Plant Sci. 13. doi: 10.3389/FPLS.2022.1087839. PMID: 36618673 PMC9815513

[B20] Fu L. Wang P. Xiong Y. (2020). Target of rapamycin signaling in plant stress responses1[open. Plant Physiol. 182, 1613–1623. doi: 10.1104/PP.19.01214. PMID: 31949028 PMC7140942

[B21] Gautam A. Pandey A. K. Dubey R. S. (2020). Unravelling molecular mechanisms for enhancing arsenic tolerance in plants: A review. Plant Gene 23, 100240. doi: 10.1016/J.PLGENE.2020.100240. PMID: 38826717

[B22] Gechev T. Petrov V. (2020). Reactive oxygen species and abiotic stress in plants. Int. J. Mol. Sci. 21, 7433. doi: 10.3390/IJMS21207433. PMID: 33050128 PMC7588003

[B23] Ghori N. H. Ghori T. Hayat M. Q. Imadi S. R. Gul A. Altay V. . (2019). Heavy metal stress and responses in plants. Int. J. Environ. Sci. Technol. 16, 1807–1828. doi: 10.1007/S13762-019-02215-8. PMID: 30311153

[B24] Gu S. Wang X. Bai J. Wei T. Sun M. Zhu L. . (2021). The kinase CIPK11 functions as a positive regulator in cadmium stress response in Arabidopsis. Gene 772, 145372. doi: 10.1016/J.GENE.2020.145372. PMID: 33346096

[B25] Gui J. Samuels T. J. Grobicki K. Z. A. Teixeira F. K. (2023). Simultaneous activation of Tor and suppression of ribosome biogenesis by TRIM-NHL proteins promotes terminal differentiation. Cell Rep. 42. doi: 10.1016/j.celrep.2023.112181. PMID: 36870055 PMC7617432

[B26] Halford N. G. Hey S. J. (2009). Snf1-related protein kinases (SnRKs) act within an intricate network that links metabolic and stress signalling in plants. Biochem. J. 419, 247–259. doi: 10.1042/BJ20082408. PMID: 19309312

[B27] Han C. Wang H. Shi W. Bai M. Y. (2024). The molecular associations between the SnRK1 complex and carbon/nitrogen metabolism in plants. New Crops 1, 100008. doi: 10.1016/J.NCROPS.2023.12.003. PMID: 38826717

[B28] Haq S. I. U. Shang J. Xie H. Qiu Q. S. (2022). Roles of TOR signaling in nutrient deprivation and abiotic stress. J. Plant Physiol. 274, 153716. doi: 10.1016/J.JPLPH.2022.153716. PMID: 35597106

[B29] Hasan M. M. Liu X. D. Waseem M. Guang-Qian Y. Alabdallah N. M. Jahan M. S. . (2022). ABA activated SnRK2 kinases: an emerging role in plant growth and physiology. Plant Signal. Behav. 17. doi: 10.1080/15592324.2022.2071024. PMID: 35506344 PMC9090293

[B30] Hosiner D. Lempiainen H. Reiter W. Urban J. Loewith R. Ammerer G. . (2008). Arsenic toxicity to Saccharomyces cerevisiae is a consequence of inhibition of the TORC1 kinase combined with a chronic stress response 1048–1057. doi: 10.1091/MBC.E08-04-0438 PMC2633375 19073887

[B31] Huang W. Zhang C. Zhu B. Liu X. Xiao H. Liu S. . (2025). Systematic evaluation of plant metals/metalloids accumulation efficiency: a global synthesis of bioaccumulation and translocation factors. Front. Plant Sci. 16, 1602951. doi: 10.3389/FPLS.2025.1602951. PMID: 40538879 PMC12178126

[B32] Jamsheer K M. Jindal S. Sharma M. Awasthi P. S S. Sharma M. . (2022). A negative feedback loop of TOR signaling balances growth and stress-response trade-offs in plants. Cell Rep. 39, 110631. doi: 10.1016/J.CELREP.2022.110631. PMID: 35385724

[B33] Jamsheer M. K. Jindal S. Laxmi A. (2019). Evolution of TOR-SnRK dynamics in green plants and its integration with phytohormone signaling networks. J. Exp. Bot. 70, 2239–2259. doi: 10.1093/JXB/ERZ107. PMID: 30870564

[B34] Jamsheer M. K. Shukla B. N. Jindal S. Gopan N. Mannully C. T. Laxmi A. (2018). The FCS-like zinc finger scaffold of the kinase SnRK1 is formed by the coordinated actions of the FLZ domain and intrinsically disordered regions. J. Biol. Chem. 293, 13134–13150. doi: 10.1074/JBC.RA118.002073. PMID: 29945970 PMC6109914

[B35] Jiang B. Liu Y. Niu H. He Y. Ma D. Li Y. (2022). Mining the roles of wheat (Triticum aestivum) SnRK genes in biotic and abiotic responses. Front. Plant Sci. 13, 934226. doi: 10.3389/FPLS.2022.934226. PMID: 35845708 PMC9280681

[B36] Kaur N. Qadir M. Francis D. V. Alok A. Tiwari S. Ahmed Z. F. R. (2025). CRISPR/Cas9: a sustainable technology to enhance climate resilience in major staple crops. Front. Genome Ed. 7. doi: 10.3389/FGEED.2025.1533197. PMID: 40171546 PMC11958969

[B37] Keyster M. Niekerk L. A. Basson G. Carelse M. Bakare O. Ludidi N. . (2020). Decoding heavy metal stress signalling in plants: Towards improved food security and safety. Plants 9, 1781. doi: 10.3390/PLANTS9121781. PMID: 33339160 PMC7765602

[B38] Khan M. I. R. Chopra P. Chhillar H. Ahanger M. A. Hussain S. J. Maheshwari C. (2021). Regulatory hubs and strategies for improving heavy metal tolerance in plants: Chemical messengers, omics and genetic engineering. Plant Physiol. Biochem. 164, 260–278. doi: 10.1016/J.PLAPHY.2021.05.006. PMID: 34020167

[B39] Kotnala S. Tiwari S. Nayak A. Bhushan B. Chandra S. Medeiros C. R. . (2025). Impact of heavy metal toxicity on the human health and environment. Sci. Total Environ. 987, 179785. doi: 10.1016/J.SCITOTENV.2025.179785. PMID: 40466229

[B40] Kulik A. Anielska-Mazur A. Bucholc M. Koen E. Szymańska K. Zmieńko A. . (2012). SNF1-related protein kinases type 2 are involved in plant responses to cadmium stress. Plant Physiol. 160, 868. doi: 10.1104/PP.112.194472. PMID: 22885934 PMC3461561

[B41] Kulik A. Wawer I. Krzywińska E. Bucholc M. Dobrowolska G. (2011). SnRK2 protein kinases—key regulators of plant response to abiotic stresses. OMICS 15, 859. doi: 10.1089/OMI.2011.0091. PMID: 22136638 PMC3241737

[B42] Li G. Zhao Y. (2024). The critical roles of three sugar-related proteins (HXK, SnRK1, TOR) in regulating plant growth and stress responses. Hortic. Res. 11. doi: 10.1093/HR/UHAE099. PMID: 38863993 PMC11165164

[B43] Li W. Liu J. Li Z. Ye R. Chen W. Huang Y. . (2024). Mitigating growth-stress tradeoffs via elevated TOR signaling in rice. Mol. Plant 17, 240–257. doi: 10.1016/j.molp.2023.12.002. PMID: 38053337 PMC11271712

[B44] Lim C. W. Lee S. C. (2023). Arabidopsis SnRK2.3/SRK2I plays a positive role in seed germination under cold stress conditions. Environ. Exp. Bot. 212, 105399. doi: 10.1016/J.ENVEXPBOT.2023.105399

[B45] Liu A. Zhou Z. Yi Y. Chen G. (2020). Transcriptome analysis reveals the roles of stem nodes in cadmium transport to rice grain. BMC Genomics 21, 1. doi: 10.1186/S12864-020-6474-7. PMID: 32028884 PMC7003353

[B46] Liu Y. Hu J. Duan X. Ding W. Xu M. Xiong Y. (2025). Target of Rapamycin (TOR): A master regulator in plant growth, development, and stress responses. Annu. Rev. Plant Biol. 76, 341–371. doi: 10.1146/annurev-arplant-083123-050311. PMID: 39952681

[B47] Luan S. (2009). The CBL-CIPK network in plant calcium signaling. Trends Plant Sci. 14, 37–42. doi: 10.1016/j.tplants.2008.10.005. PMID: 19054707

[B48] Ma X. Li Q. H. Yu Y. N. Qiao Y. M. Haq S. U. Gong Z. H. (2020). The CBL–CIPK pathway in plant response to stress signals. Int. J. Mol. Sci. 21, 5668. doi: 10.3390/IJMS21165668. PMID: 32784662 PMC7461506

[B49] Manna M. Rengasamy B. Sinha A. K. (2023). Revisiting the role of MAPK signalling pathway in plants and its manipulation for crop improvement. Plant Cell Environ. 46, 2277–2295. doi: 10.1111/PCE.14606. PMID: 37157977

[B50] Mansoor S. Ali A. Kour N. Bornhorst J. AlHarbi K. Rinklebe J. . (2023). Heavy metal induced oxidative stress mitigation and ROS scavenging in plants. Plants 12, 3003. doi: 10.3390/PLANTS12163003. PMID: 37631213 PMC10459657

[B51] Margalha L. Confraria A. Baena-González E. (2019). SnRK1 and TOR: modulating growth–defense trade-offs in plant stress responses. J. Exp. Bot. 70, 2261–2274. doi: 10.1093/jxb/erz066. PMID: 30793201

[B52] Meng Y. Zhang N. Li J. Shen X. Sheen J. Xiong Y. (2022). TOR kinase, a GPS in the complex nutrient and hormonal signaling networks to guide plant growth and development. J. Exp. Bot. 73, 7041. doi: 10.1093/JXB/ERAC282. PMID: 35781569 PMC9664236

[B53] Mishra R. K. Mentha S. S. Misra Y. Dwivedi N. (2023). Emerging pollutants of severe environmental concern in water and wastewater: a comprehensive review on current developments and future research. Water-Energy Nexus 6, 74–95. doi: 10.1016/J.WEN.2023.08.002. PMID: 38826717

[B54] Mitra M. Agarwal P. Roy S. (2023). “ Plant response to heavy metal stress: an insight into the molecular mechanism of transcriptional regulation,” in Plant Transcription Factors: Contribution in Development, Metabolism, and Environmental Stress. (Cambridge, MA, USA: Elsevier), 337–367. doi: 10.1016/B978-0-323-90613-5.00004-2

[B55] Mohamed H. I. Ullah I. Toor M. D. Tanveer N. A. Din M. M. U. Basit A. . (2025). Heavy metals toxicity in plants: understanding mechanisms and developing coping strategies for remediation: a review. Bioresources Bioprocessing 12, 1. doi: 10.1186/S40643-025-00930-4. PMID: 40906247 PMC12411398

[B56] Mondal S. Paul A. Mitra D. Pradhan C. Seth C. S. Chattopadhyay K. . (2024). The multifaceted role of different SnRK gene family members in regulating multiple abiotic stresses in plants. Physiol. Plant 176. doi: 10.1111/PPL.14543. PMID: 39403065

[B57] Morales-Herrera S. Paul M. J. Van Dijck P. Beeckman T. (2024). SnRK1/TOR/T6P: three musketeers guarding energy for root growth. Trends Plant Sci. 29, 1066–1076. doi: 10.1016/J.TPLANTS.2024.03.006. PMID: 38580543

[B58] Munteanu C. Turnea M. A. Rotariu M. (2023). Hydrogen sulfide: an emerging regulator of oxidative stress and cellular homeostasis—a comprehensive one-year review. Antioxidants 12, 1737. doi: 10.3390/ANTIOX12091737. PMID: 37760041 PMC10526107

[B59] Naaz S. Ahmad N. Al-Huqail A. A. Irfan M. Khan F. Qureshi M. I. (2023a). Cd and Hg mediated oxidative stress, antioxidative metabolism and molecular changes in soybean (Glycine max L.). Phyton (B. Aires). 92, 1725–1742. doi: 10.32604/PHYTON.2023.026100

[B60] Naaz S. Ahmad N. Jameel M. R. Al-Huqail A. A. Khan F. Qureshi M. I. (2023b). Impact of some toxic metals on important ABC transporters in soybean (Glycine max L.). ACS Omega 8, 27597–27611. doi: 10.1021/ACSOMEGA.3C03325. PMID: 37546587 PMC10399161

[B61] Ni S. Qu H. Liu D. Yuan L. Li M. Zhao W. (2025). Recent advances of the mechanistic target of rapamycin (mTOR) inhibitors for drug discovery. Eur. J. Med. Chem. 298, 118046. doi: 10.1016/J.EJMECH.2025.118046. PMID: 40784306

[B62] Niekerk L. A. Gokul A. Basson G. Badiwe M. Nkomo M. Klein A. . (2024). Heavy metal stress and mitogen activated kinase transcription factors in plants: exploring heavy metal-ROS influences on plant signalling pathways. Plant Cell Environ. 47, 2793–2810. doi: 10.1111/PCE.14926. PMID: 38650576

[B63] Oraby H. F. Elnaggar N. Z. Omar A. A. Mohamed A. H. (2025). Role of serotonin in cadmium mitigation in plants. Plants 14, 1738. doi: 10.3390/PLANTS14121738. PMID: 40573726 PMC12196318

[B64] Page V. Feller U. (2015). Heavy metals in crop plants: transport and redistribution processes on the whole plant level. Agronomy 5, 447–463. doi: 10.3390/AGRONOMY5030447. PMID: 30654563

[B65] Parwez R. Aftab T. Khan M. M. A. Naeem M. (2023). Exogenous abscisic acid fine-tunes heavy metal accumulation and plant’s antioxidant defence mechanism to optimize crop performance and secondary metabolite production in Trigonella foenum-graecum L. under nickel stress. Plant Sci. 332, 111703. doi: 10.1016/J.PLANTSCI.2023.111703. PMID: 37031743

[B66] Peixoto B. Baena-González E. (2022). Management of plant central metabolism by SnRK1 protein kinases. J. Exp. Bot. 73, 7068. doi: 10.1093/JXB/ERAC261. PMID: 35708960 PMC9664233

[B67] Pu Y. Luo X. Bassham D. C. (2017). Tor-dependent and -independent pathways regulate autophagy in arabidopsis thaliana. Front. Plant Sci. 8, 1204. doi: 10.3389/FPLS.2017.01204/FULL 28744293 PMC5504165

[B68] Rabeh K. Sallami A. Gaboun F. Filali-Maltouf A. Sbabou L. Belkadi B. (2024). Genome-wide analysis of aquaporin and their responses to abiotic stresses in plants: a systematic review and meta-analysis. Plant Stress 11, 100362. doi: 10.1016/J.STRESS.2024.100362. PMID: 38826717

[B69] Rahman Z. Pal Singh V. Rahman Z. Singh V. P. (2019). The relative impact of toxic heavy metals (THMs) (arsenic (As), cadmium (Cd), chromium (Cr)(VI), mercury (Hg), and lead (Pb)) on the total environment: an overview. Environ. Monit. Assess. 191, 7. doi: 10.1007/S10661-019-7528-7. PMID: 31177337

[B70] Rai P. K. Lee S. S. Zhang M. Tsang Y. F. Kim K. H. (2019). Heavy metals in food crops: health risks, fate, mechanisms, and management. Environ. Int. 125, 365–385. doi: 10.1016/J.ENVINT.2019.01.067. PMID: 30743144

[B71] Riaz M. Kamran M. Fang Y. Wang Q. Cao H. Yang G. . (2021). Arbuscular mycorrhizal fungi-induced mitigation of heavy metal phytotoxicity in metal contaminated soils: a critical review. J. Hazard. Mater. 402, 123919. doi: 10.1016/J.JHAZMAT.2020.123919. PMID: 33254825

[B72] Rodriguez M. Parola R. Andreola S. Pereyra C. Martínez-Noël G. (2019). TOR and SnRK1 signaling pathways in plant response to abiotic stresses: do they always act according to the “yin-yang” model? Plant Sci. 288, 110220. doi: 10.1016/J.PLANTSCI.2019.110220. PMID: 31521220

[B73] Rohde J. Heitman J. Cardenas M. E. (2001). The TOR kinases link nutrient sensing to cell growth. J. Biol. Chem. 276, 9583–9586. doi: 10.1074/JBC.R000034200. PMID: 11266435

[B74] Saile J. Wießner-Kroh T. Erbstein K. Obermüller D. M. Pfeiffer A. Janocha D. . (2023). SNF1-RELATED KINASE 1 and TARGET OF RAPAMYCIN control light-responsive splicing events and developmental characteristics in etiolated Arabidopsis seedlings. Plant Cell 35, 3413–3428. doi: 10.1093/PLCELL/KOAD168. PMID: 37338062 PMC10473197

[B75] Sen B. Roy D. Narayan M. Sarma H. (2025). Nanoparticle-driven stress alleviation: exploring the roles of metal and metal oxide nanoparticles in plant abiotic stress management. Discover Plants 2, 1. doi: 10.1007/S44372-025-00153-Z. PMID: 30311153

[B76] Sharma M. Sharma M. Jamsheer M. K. Laxmi A. (2022). A glucose-target of rapamycin signaling axis integrates environmental history of heat stress through maintenance of transcription-associated epigenetic memory in Arabidopsis. J. Exp. Bot. 73, 7083–7102. doi: 10.1093/JXB/ERAC338. PMID: 35980748

[B77] Sharma N. Negi N. Kaur H. Sharma V. Kumari V. Thakur N. (2025). Metabolic adaptations in plants: navigating heavy metal stress for sustainable plant growth. Discover Appl. Sci. 7, 10. doi: 10.1007/S42452-025-06715-W. PMID: 30311153

[B78] Shetty B. R. Jagadeesha P. B. Salmataj S. A. (2025). Heavy metal contamination and its impact on the food chain: exposure, bioaccumulation, and risk assessment. CyTA J. Food 23. doi: 10.1080/19476337.2024.2438726. PMID: 37339054

[B79] Shi L. Wu Y. Sheen J. (2018). TOR signaling in plants: conservation and innovation. Development 145, dev160887. doi: 10.1242/DEV.160887. PMID: 29986898 PMC6053665

[B80] Shi X. Bao J. Lu X. Ma L. Zhao Y. Lan S. . (2023). The mechanism of Ca2+ signal transduction in plants responding to abiotic stresses. Environ. Exp. Bot. 216, 105514. doi: 10.1016/J.ENVEXPBOT.2023.105514. PMID: 38826717

[B81] Shu H. Zhang Y. Liu J. Zhang L. Lu X. Liu Z. . (2026). Molecular characterization of SnRK2 gene family in Capsicum chinense and functional validation of CcSnRK2.5 under drought stress. Plant Cell Rep. 45, 84. doi: 10.1007/s00299-026-03766-0. PMID: 41811511

[B82] Son S. Park S. R. (2023). The rice SnRK family: biological roles and cell signaling modules. Front. Plant Sci. 14. doi: 10.3389/FPLS.2023.1285485 PMC10644236 38023908

[B83] Soto-Burgos J. Bassham D. C. (2017). SnRK1 activates autophagy via the TOR signaling pathway in Arabidopsis thaliana. PloS One 12, e0182591. doi: 10.1371/JOURNAL.PONE.0182591. PMID: 28783755 PMC5544219

[B84] Szymańska K. P. Polkowska-Kowalczyk L. Lichocka M. Maszkowska J. Dobrowolska G. (2019). SNF1-related protein kinases SnRK2.4 and SnRK2.10 modulate ROS homeostasis in plant response to salt stress. Int. J. Mol. Sci. 20. doi: 10.3390/IJMS20010143. PMID: 30609769 PMC6337402

[B85] Tang R. J. Wang C. Li K. Luan S. (2020). The CBL–CIPK calcium signaling network: unified paradigm from 20 years of discoveries. Trends Plant Sci. 25, 604–617. doi: 10.1016/J.TPLANTS.2020.01.009. PMID: 32407699

[B86] Tiwari S. Lata C. (2018). Heavy metal stress, signaling, and tolerance due to plant-associated microbes: an overview. Front. Plant Sci. 9, 336111. doi: 10.3389/FPLS.2018.00452/FULL PMC5897519 29681916

[B87] Tripathi R. D. Tripathi P. Dwivedi S. Dubey S. Chatterjee S. Chakrabarty D. . (2012). Arsenomics: omics of arsenic metabolism in plants. Front. Physiol. 3, 275. doi: 10.3389/FPHYS.2012.00275. PMID: 22934029 PMC3429049

[B88] Umar A. W. Naeem M. Hussain H. Ahmad N. Xu M. (2025). Starvation from within: how heavy metals compete with essential nutrients, disrupt metabolism, and impair plant growth. Plant Sci. 353, 112412. doi: 10.1016/J.PLANTSCI.2025.112412. PMID: 39920911

[B89] Viehweger K. (2014). How plants cope with heavy metals. Bot. Stud. 55, 35. doi: 10.1186/1999-3110-55-35/TABLES/3 28510963 PMC5432744

[B90] Vitelli V. Giamborino A. Bertolini A. Saba A. Andreucci A. (2024). Cadmium stress signaling pathways in plants: molecular responses and mechanisms. Curr. Issues Mol. Biol. 46, 6052–6068. doi: 10.3390/CIMB46060361. PMID: 38921032 PMC11202648

[B91] Wang P. Zhao Y. Li Z. Hsu C. C. Liu X. Fu L. . (2018). Reciprocal regulation of the TOR kinase and ABA receptor balances plant growth and stress response. Mol. Cell 69, 100–112.e6. doi: 10.1016/J.MOLCEL.2017.12.002. PMID: 29290610 PMC5772982

[B92] Wullschleger S. Loewith R. Hall M. N. (2006). TOR signaling in growth and metabolism. Cell. 124, 471–484. doi: 10.1016/J.CELL.2006.01.016. PMID: 16469695

[B93] Xiong Y. McCormack M. Li L. Hall Q. Xiang C. Sheen J. (2013). Glucose-TOR signalling reprograms the transcriptome and activates meristems. Nature 496, 181–186. doi: 10.1038/NATURE12030. PMID: 23542588 PMC4140196

[B94] Xu L. Zheng Y. Yu Q. Liu J. Yang Z. Chen Y. (2022). Transcriptome analysis reveals the stress tolerance to and accumulation mechanisms of cadmium in Paspalum vaginatum Swartz. Plants 11, 2078. doi: 10.3390/PLANTS11162078/S1 36015382 PMC9414793

[B95] Xu Q. Kong F. Yang W. (2025). SnRK1 as the core node integrating energy homoeostasis, stress adaptation and hormonal crosstalk in plants. Plant Cell Environ. 48. doi: 10.1111/PCE.70074. PMID: 40692523

[B96] Xu Y. Fu X. (2022). Reprogramming of plant central metabolism in response to abiotic stresses: a metabolomics view. Int. J. Mol. Sci. 23, 5716. doi: 10.3390/IJMS23105716. PMID: 35628526 PMC9143615

[B97] Yang C. Li X. Yang L. Chen S. Liao J. Li K. . (2023). A positive feedback regulation of SnRK1 signaling by autophagy in plants. Mol. Plant 16, 1192–1211. doi: 10.1016/j.molp.2023.07.001. PMID: 37408307

[B98] Yang G. Yu Z. Gao L. Zheng C. (2019). SnRK2s at the crossroads of growth and stress responses. Trends Plant Sci. 24, 672–676. doi: 10.1016/J.TPLANTS.2019.05.010. PMID: 31255544

[B99] Yang L. Zhang R. Zhang H. Yang Y. Fu L. (2025). TOR mediates stress responses through global regulation of metabolome in plants. Int. J. Mol. Sci. 26. doi: 10.3390/ijms26052095. PMID: 40076716 PMC11900525

[B100] Yang M. Lu Y. Piao W. Jin H. (2022). The Translational Regulation in mTOR Pathway. Biomolecules 12, 802. doi: 10.3390/BIOM12060802 35740927 PMC9221026

[B101] Yu X. Yang L. Fan C. Hu J. Zheng Y. Wang Z. . (2023). Abscisic acid (ABA) alleviates cadmium toxicity by enhancing the adsorption of cadmium to root cell walls and inducing antioxidant defense system of Cosmos bipinnatus. Ecotoxicol. Environ. Saf. 261, 115101. doi: 10.1016/J.ECOENV.2023.115101. PMID: 37290296

[B102] Zhang H. Mao X. Wang C. Jing R. (2010). Overexpression of a common wheat gene TaSnRK2.8 enhances tolerance to drought, salt and low temperature in Arabidopsis. PloS One 5, e16041. doi: 10.1371/journal.pone.0016041. PMID: 21209856 PMC3012728

[B103] Zhao R. Yin K. Chen S. (2022). Hydrogen sulphide signalling in plant response to abiotic stress. Plant Biol. (Stuttg). 24, 523–531. doi: 10.1111/PLB.13367. PMID: 34837449

[B104] Zhao Y. Wang J. Huang W. Zhang D. Wu J. Li B. . (2023). Abscisic-acid-regulated responses to alleviate cadmium toxicity in plants. Plants 12, 1023. doi: 10.3390/PLANTS12051023. PMID: 36903884 PMC10005406

[B105] Zhao Y. Wu Z. Zhang J. Li G. (2026). Crosstalk and coordination of TOR, SnRK1, and ABA in plant metabolic and environmental adaptation. Plant Sci. 364, 112965. doi: 10.1016/j.plantsci.2025.112965. PMID: 41461324

[B106] Zheng R. Zhao K. Chen J. Zhu X. Peng Y. Shen M. . (2025). Genomic signatures of SnRKs highlighted conserved evolution within orchids and stress responses through ABA signaling in the Cymbidium ensifolium. BMC Plant Biol. 25. doi: 10.1186/S12870-025-06280-9. PMID: 40025443 PMC11874761

[B107] Zhou P. Adeel M. Shakoor N. Guo M. Hao Y. Azeem I. . (2020). Application of nanoparticles alleviates heavy metals stress and promotes plant growth: An overview. Nanomaterials 11, 26. doi: 10.3390/NANO11010026. PMID: 33374410 PMC7824443

[B108] Zhu W. Wu D. Jiang L. Ye L. (2020). Genome-wide identification and characterization of SnRK family genes in Brassica napus. BMC Plant Biol. 20, 1–14. doi: 10.1186/S12870-020-02484-3/FIGURES/9 32571241 PMC7310057

